# Supplementary material for: CO-tolerant RuNi/TiO2 catalyst for the storage and purification of crude hydrogen
Source: Nat Commun. 2022 Jul 29;13:4404. doi: 10.1038/s41467-022-32100-x (PMC9338308; doi:10.1038/s41467-022-32100-x)
Supplement: Supplementary file 1 — Supplementary Information [file 41467_2022_32100_MOESM1_ESM.pdf]

Supporting Information

CO-tolerant RuNi/TiO_2 Catalyst for the Storage and Purification of Crude Hydrogen

Zhaohua Wang,[1,#] *Chunyang Dong,*[1,2,#] *Xuan Tang,*[3,#] *Xuetao Qin,*[1] *Xingwu Liu,*[1,4] *Mi Peng,*[1] *Yao Xu,*[1] *Chuqiao Song,*[1] *Jie Zhang,*[1] *Xuan Liang,*[1] *Sheng Da*[3,*] *and Ding Ma*[1,*]

[1]Beijing National Laboratory for Molecular Sciences, College of Chemistry and Molecular Engineering and College of Engineering, and BIC-ESAT, Peking University, Beijing 100871 (P. R. China)

[2]UCCS–Unité de Catalyse et Chimie du Solide, Université de Lille, CNRS, Centrale Lille, ENSCL, Université d'Artois, UMR 8181, Lille, France

[3]Key Laboratory for Advanced Materials and Joint International Research Laboratory of Precision Chemistry and Molecular Engineering, Feringa Nobel Prize Scientist Joint Research Centre, Frontiers Science Center for Materiobiology and Dynamic Chemistry, Institute of Fine Chemicals, School of Chemistry and Molecular Engineering, East China University of Science & Technology, Shanghai 200237, China

[4]National Energy Center for Coal to Liquids, Synfuels CHINA Co., Ltd., Beijing 101400, China

*Correspondence: shengdai@ecust.edu.cn (D.S.)

dma@pku.edu.cn (D.M.)

[#] These authors contributed equally to this work.

Supplementary Figures and Tables

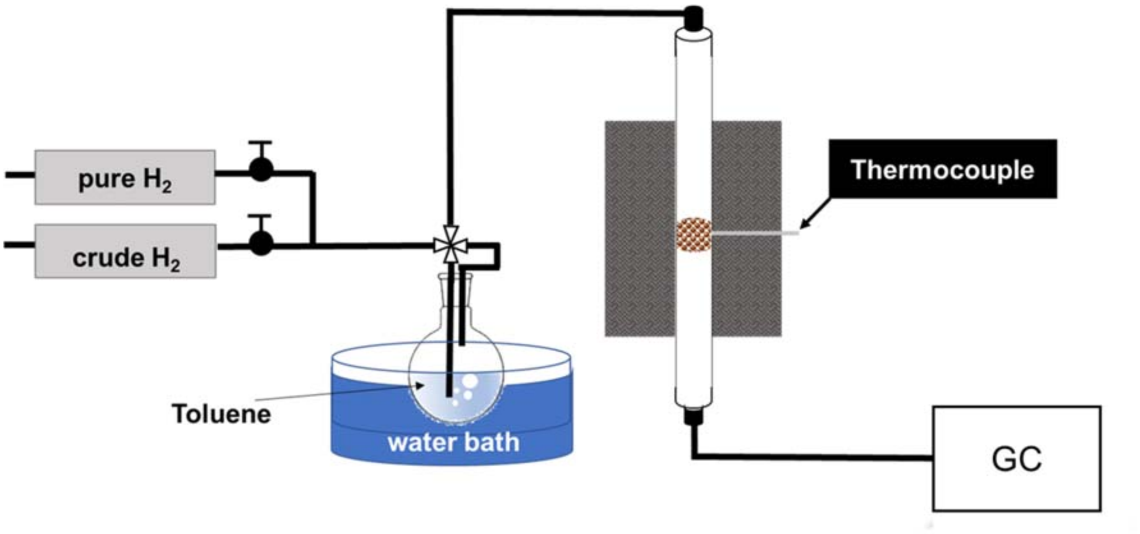

Supplementary Figure 1. Schematic illustration of the catalytic evaluation setup.

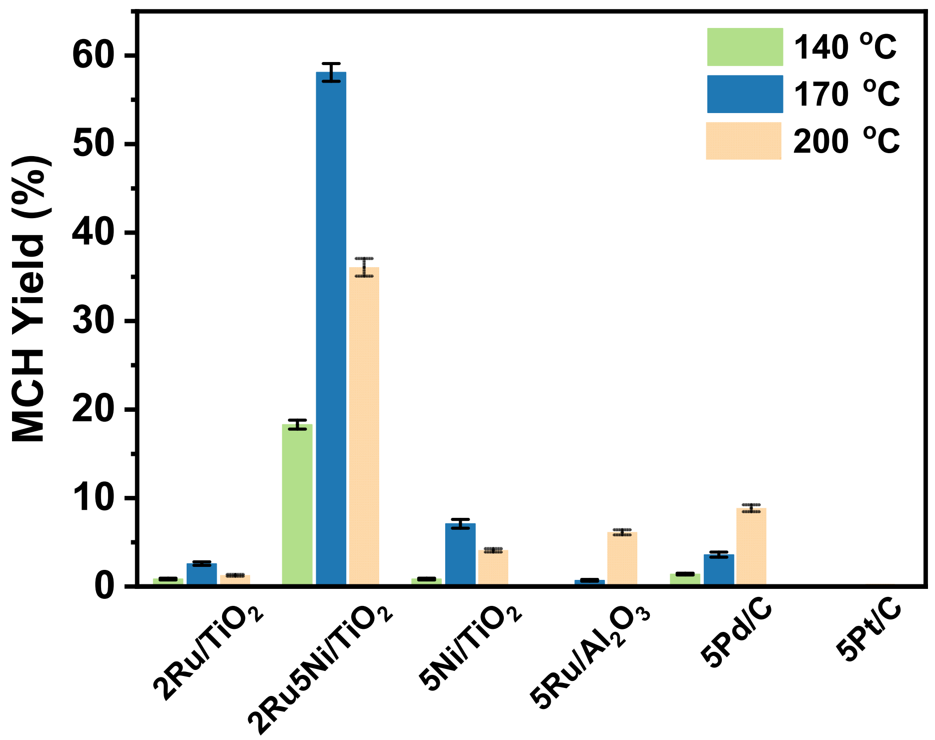

Supplementary Figure 2. MCH Yields of various catalysts of CO tolerant toluene hydrogenation reaction. Reaction conditions: 0.1 %CO/0.1 %Ar/H_2, GHSV of the carrier gas = 36,000 mL/g_{cat}/h and WHSV of toluene = 2.1 h^{-1}.

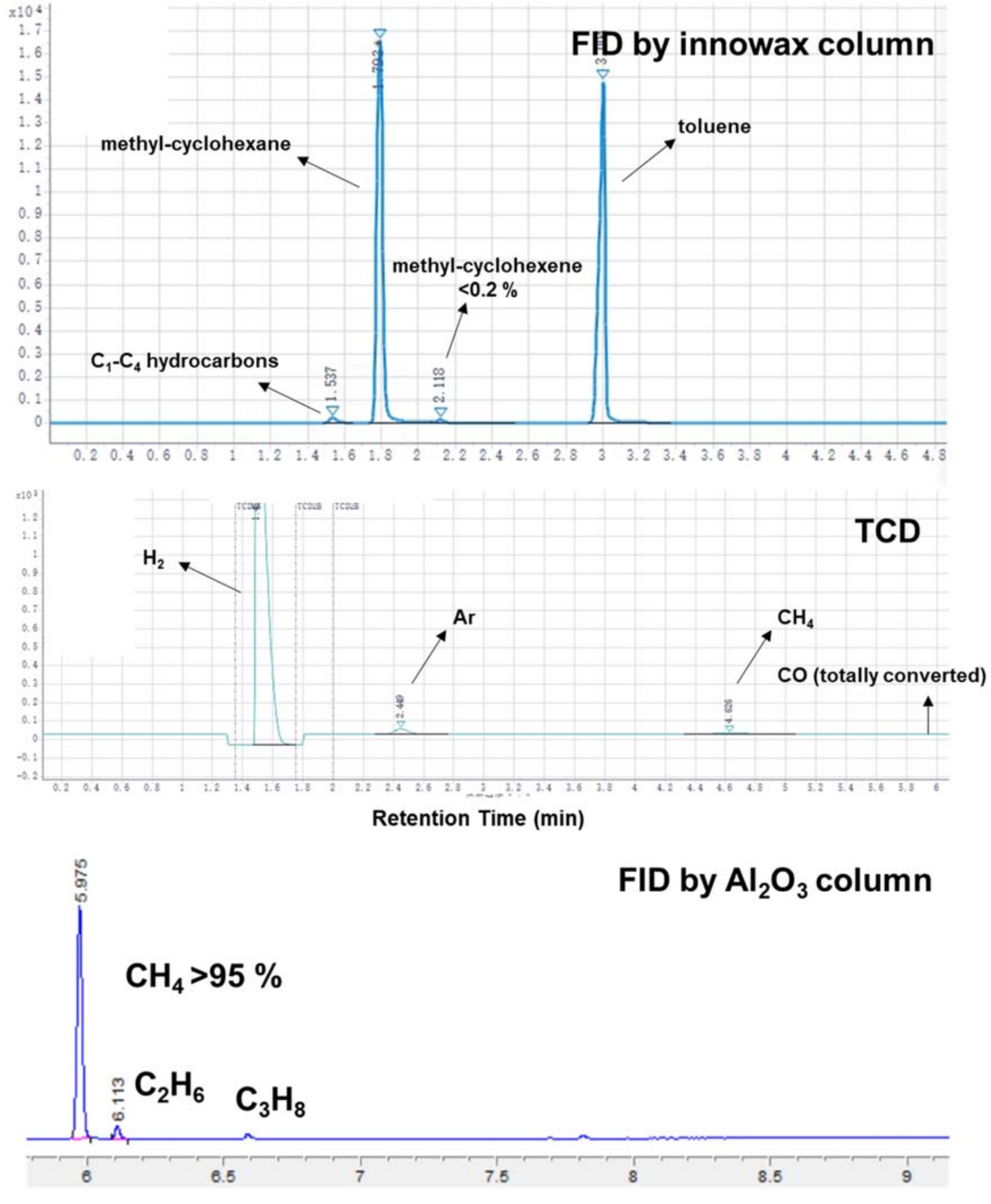

Supplementary Figure 3. Typical GC analysis graph of CO-tolerant toluene hydrogenation using 2Ru5Ni/TiO_2.

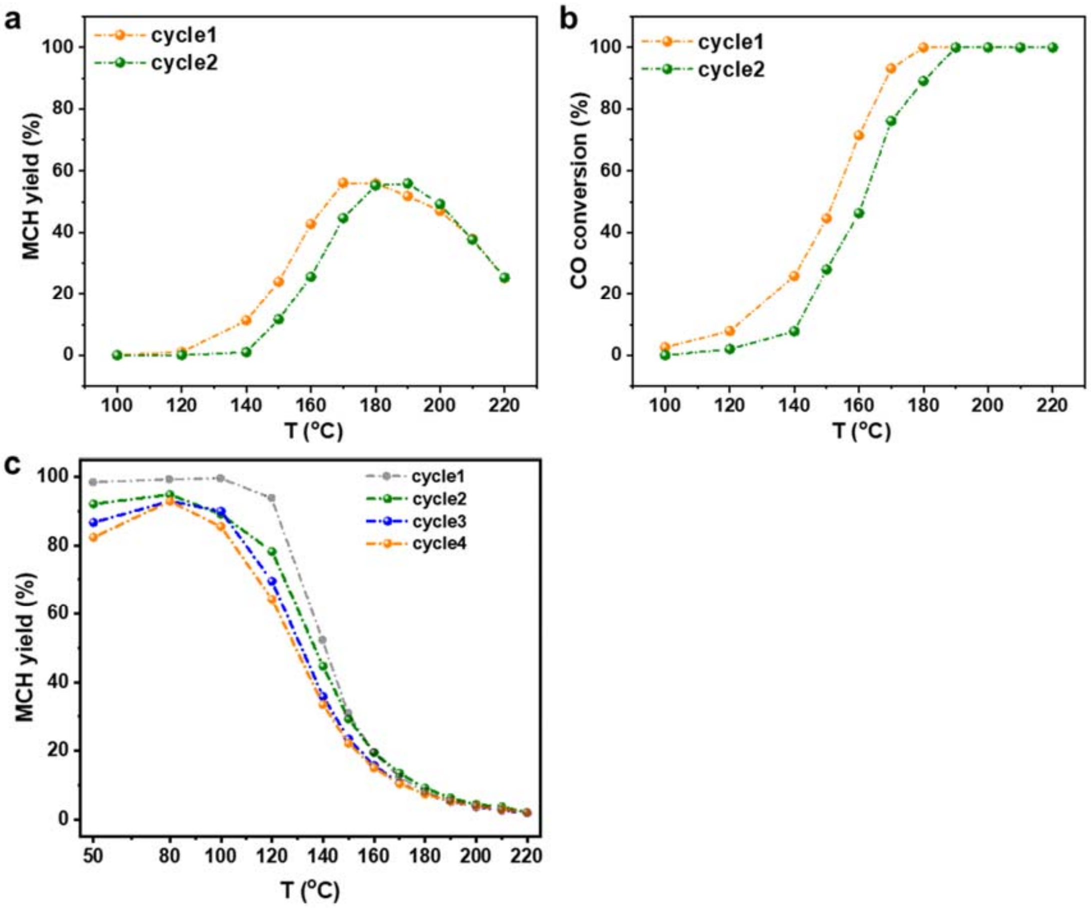

Supplementary Figure 4. Cyclicality of **2Ru5Ni/TiO_2** for CO-tolerant toluene hydrogenation reaction at 100 – 220 °C. (a) MCH yields and (b) CO conversion. Reaction conditions: 0.1 %CO/0.1 %Ar/H_2, GHSV of the carrier gas = 36,000 mL/g_{cat}/h and WHSV of toluene = 2.1 h^{-1}. Cyclicality of **2Ru/TiO_2** for pure H_2 toluene hydrogenation reaction (c) MCH yields profile at 50 – 220 °C. Reaction conditions: pure H_2, GHSV of the carrier gas = 36,000 mL/g_{cat}/h and WHSV of toluene = 2.1 h^{-1}.

Supplementary Note 1: Both 2Ru/TiO_2 and 2Ru5Ni/TiO_2 displayed similar trends in different cycles, indicating that the drop of MCH yields with temperature increasing higher than a specific temperature was not caused by irreversible structural change of the catalysts. However, after first cycle, it can be observed their activities of toluene hydrogenation slightly decreased, probably due to the Ru or Ni particles agglomeration during reaction, which was confirmed in **Supplementary Figures 10** and **18**.

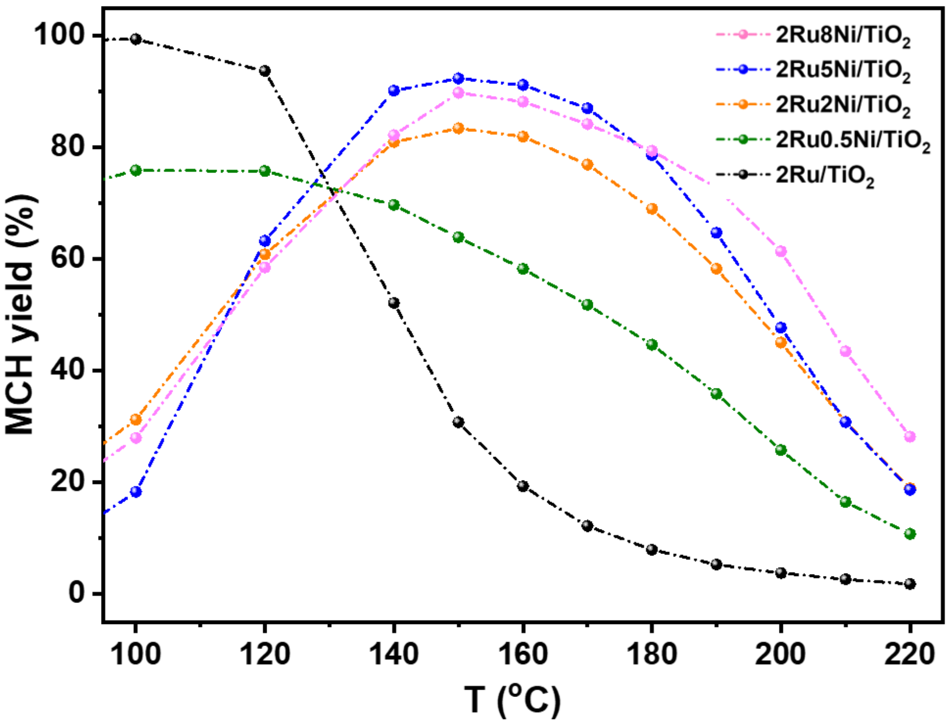

Supplementary Figure 5. Toluene hydrogenation activity for 2Ru*x*Ni/TiO_2 using pure hydrogen. Reaction conditions: GHSV of the carrier gas = 36,000 mL/g_{cat}/h and WHSV of toluene = 2.1 h^{-1}.

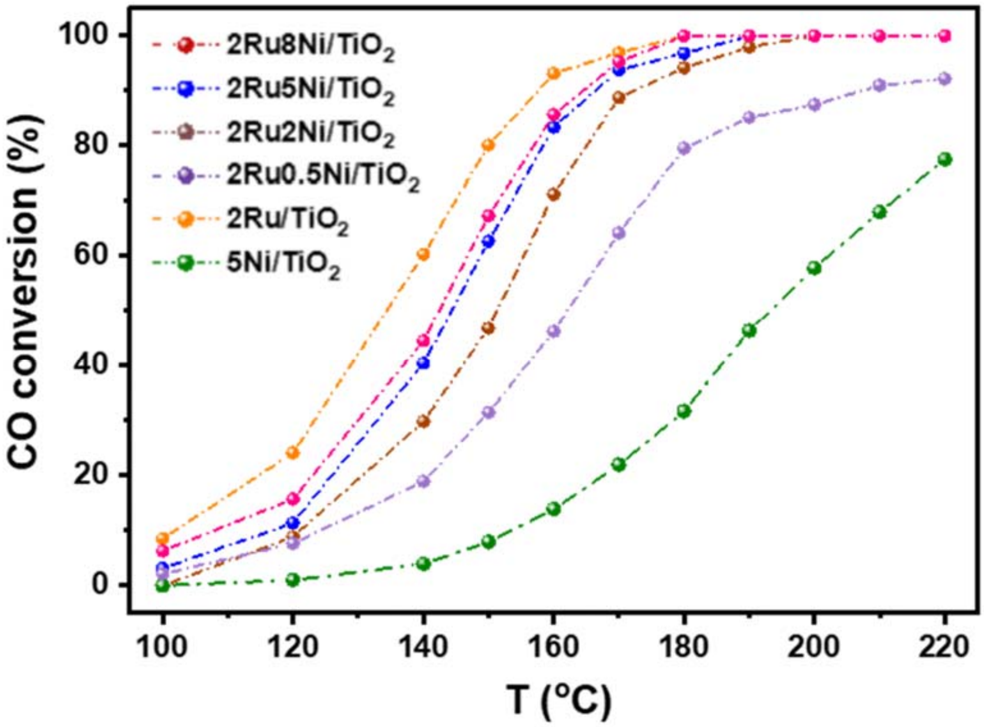

Supplementary Figure 6. CO conversion in CO-tolerant toluene hydrogenation reaction for $5Ni/TiO_2$ and $2Ru\textit{x}Ni/TiO_2$. Reaction conditions: 0.1 %CO/0.1 %Ar/H_2, GHSV of the carrier gas = 36,000 mL/g_{cat}/h and WHSV of toluene = 2.1 h^{-1}.

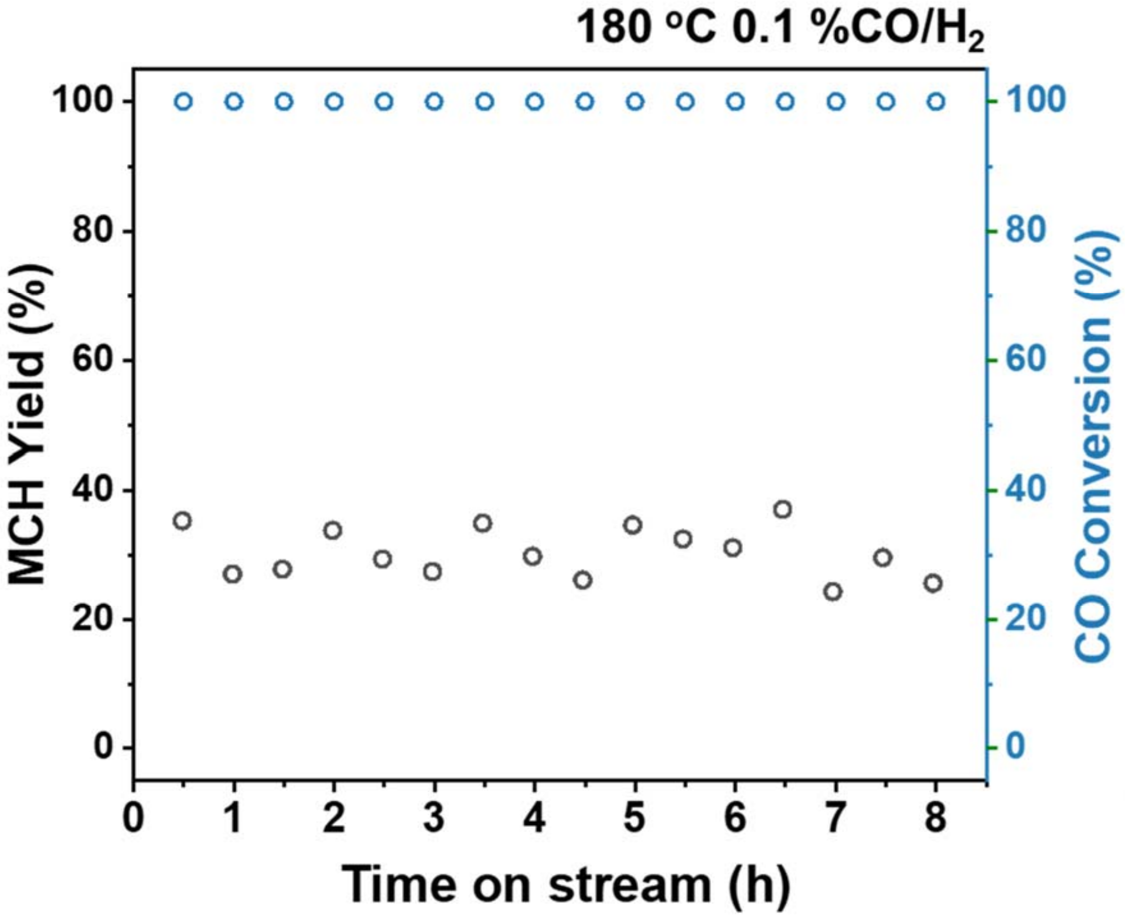

Supplementary Figure 7. Toluene hydrogenation activity over $2Ru5Ni/TiO_2$ on a trickle bed reactor using 0.1 %CO/H_2 at 180 ºC. Reaction conditions: 0.1 g catalyst, toluene feed= 0.02 mL/min, 0.1 %CO/H_2 = 50 mL/min, 180 ℃, 1 bar. The fluctuation was due to the unstable feeding.

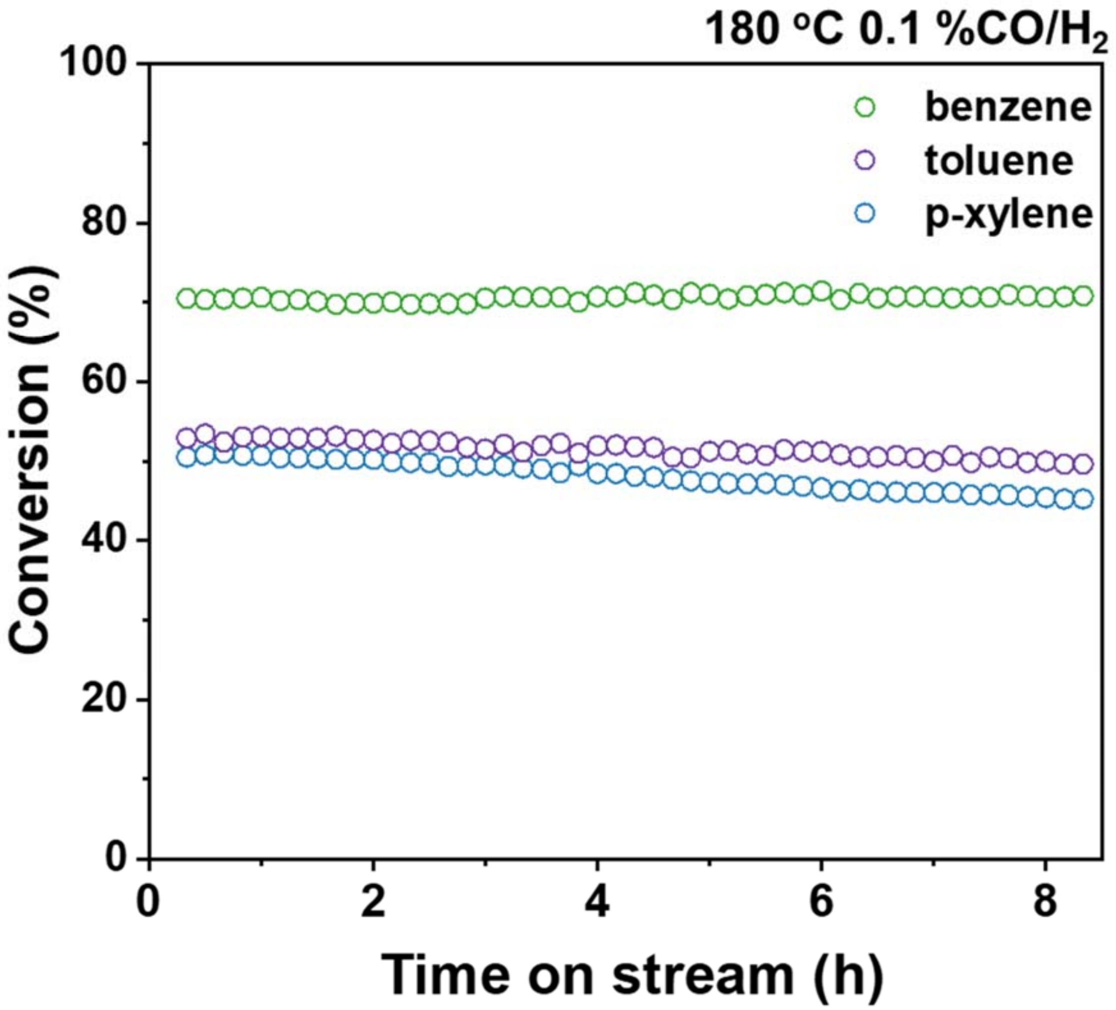

Supplementary Figure 8. CO-tolerant aromatics (benzene, toluene and p-xylene) hydrogenation activities over 2Ru5Ni/TiO_2. Reaction conditions: 180 °C, 0.1 %CO/0.1 %Ar/H_2, GHSV of the carrier gas = 36,000 mL/g_{cat}/h, WHSV of benzene = 5.3 h^{-1}, toluene = 2.1 h^{-1}, p-xylene = 1.5 h^{-1}.

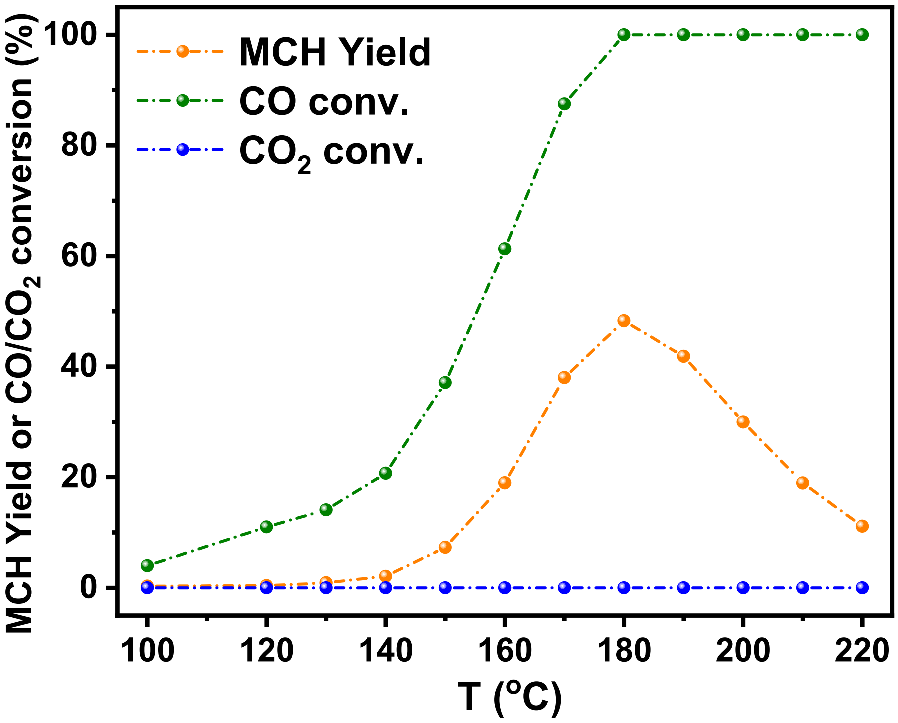

Supplementary Figure 9. CO-CO_2 containing toluene hydrogenation activity of 2Ru5Ni/TiO_2. Reaction conditions: 0.1 %CO/19 %CO_2/0.1 %Ar/H_2, GHSV of the carrier gas = 12,000 mL/g_{cat}/h and WHSV of toluene = 1.4 h^{-1}.

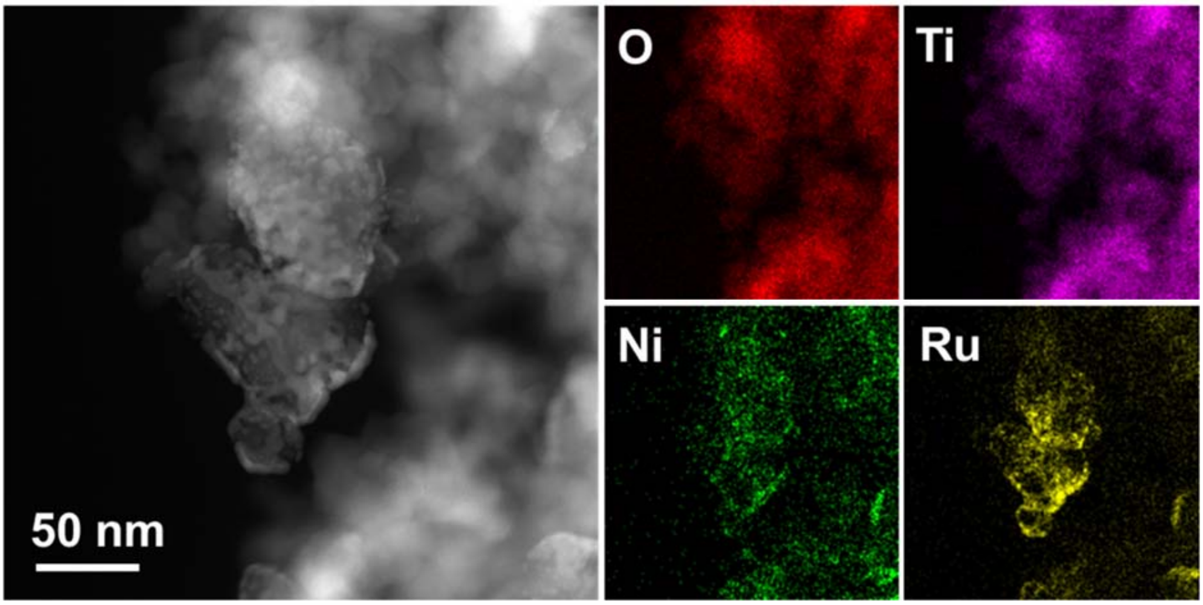

Supplementary Figure 10. Representative HAADF-STEM image and the corresponding EDS mapping images of Ni and Ru of spent $2Ru5Ni/TiO_2$ after one cycle of 100 – 220 ºC CO tolerant toluene hydrogenation reaction; Reaction condition: 0.1 %CO/0.1 %Ar/H_2, GHSV of the carrier gas = 36,000 mL/g_{cat}/h and WHSV of toluene = 2.1 h^{-1}. From the STEM results, some Ru species in $2Ru5Ni/TiO_2$ slightly agglomerated in the reaction, which induced the growth of Ni particles either.

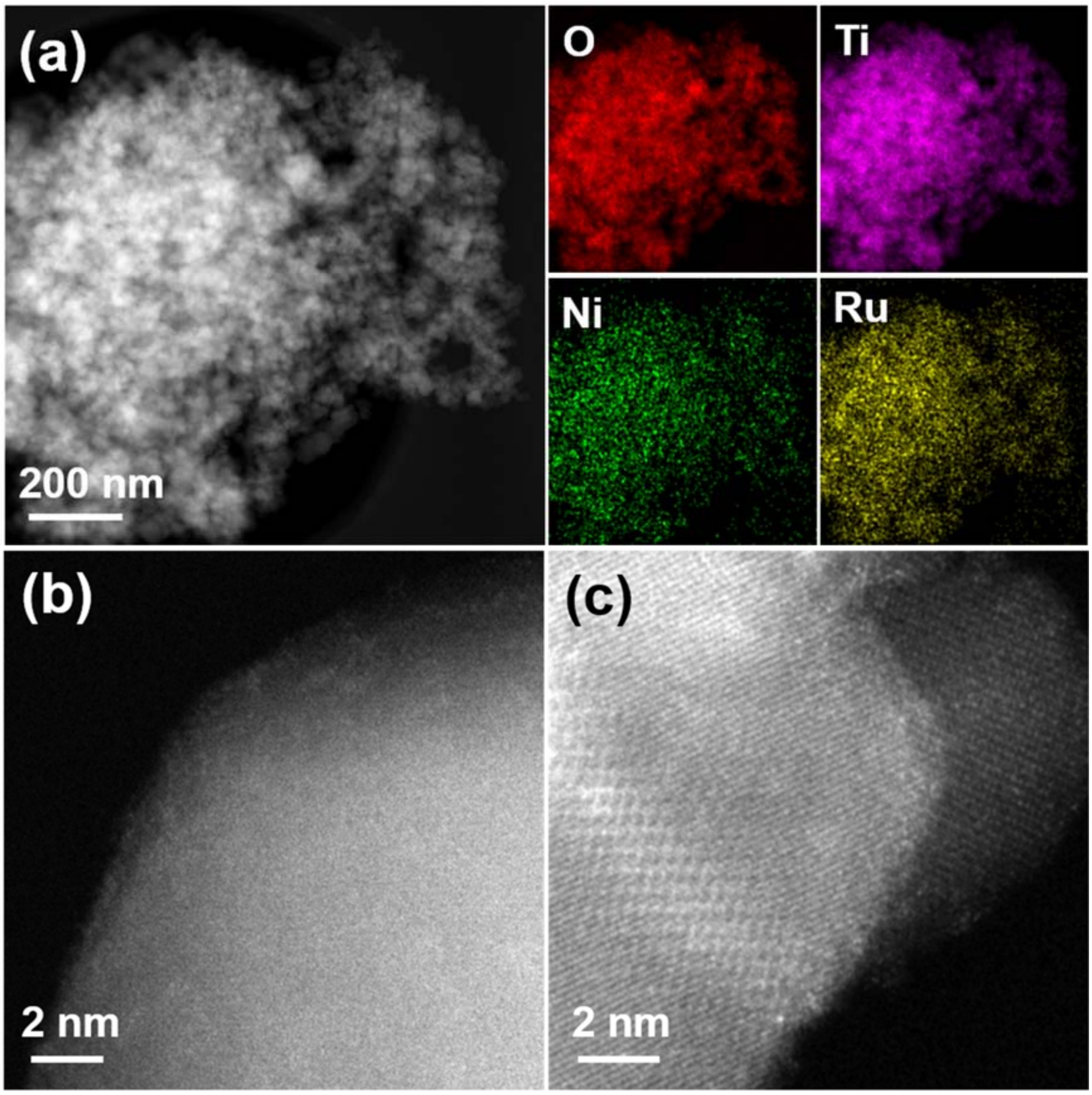

Supplementary Figure 11. Representative HAADF-STEM images and the corresponding EDS mapping images of O, Ti, Ni, and Ru of 2Ru5Ni/TiO_2 before calcination. (a) low-magnification, (b) and (c) high-magnification HAADF-STEM images of 2Ru5Ni/TiO_2.

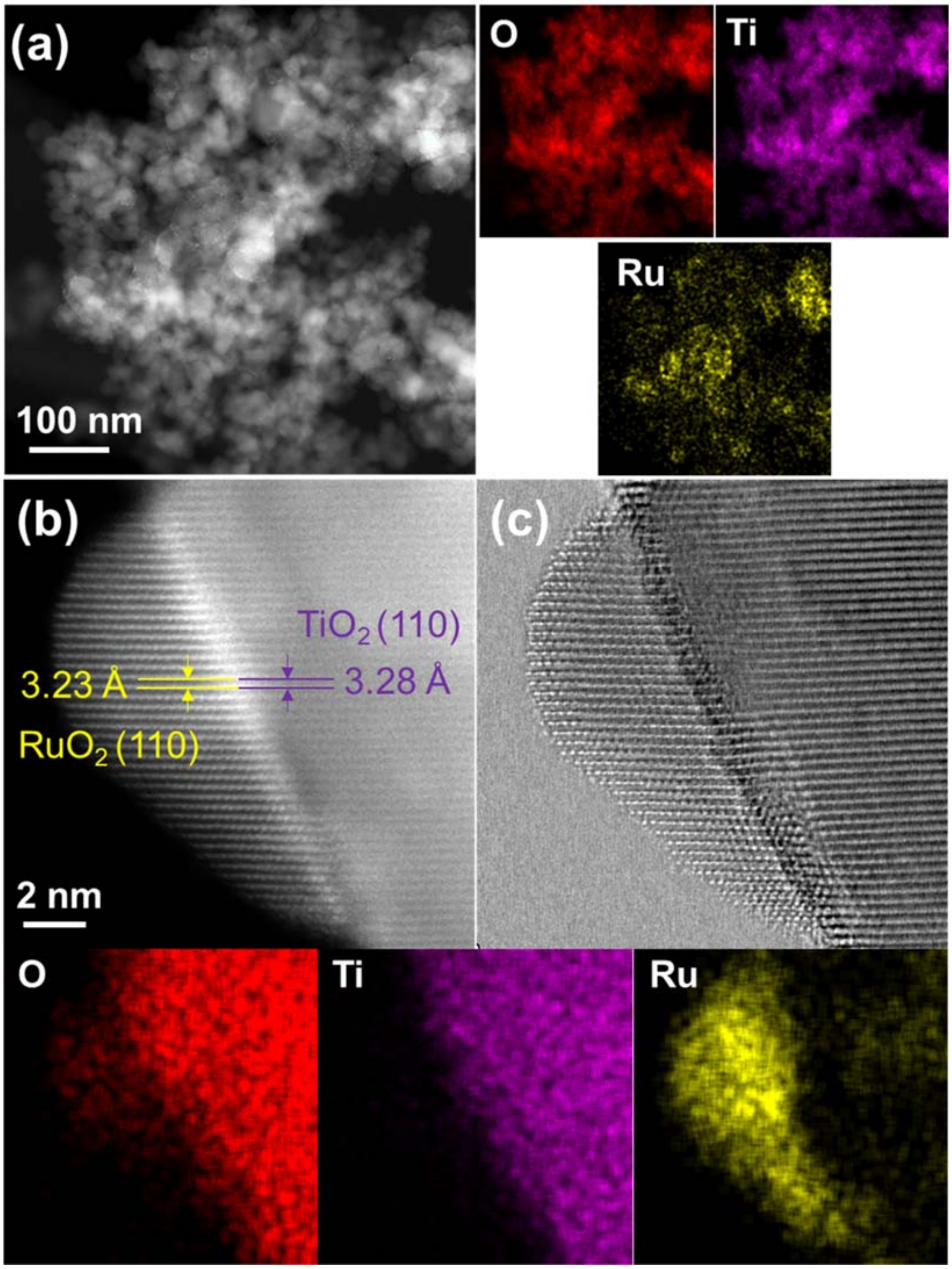

Supplementary Figure 12. Representative HAADF-STEM images of $2Ru/TiO_2$ after calcination and the corresponding EDS-mapping images of O, Ti, and Ru. (a) low-magnification, (b) and (c) high-magnification HAADF-STEM images of $2Ru/TiO_2$.

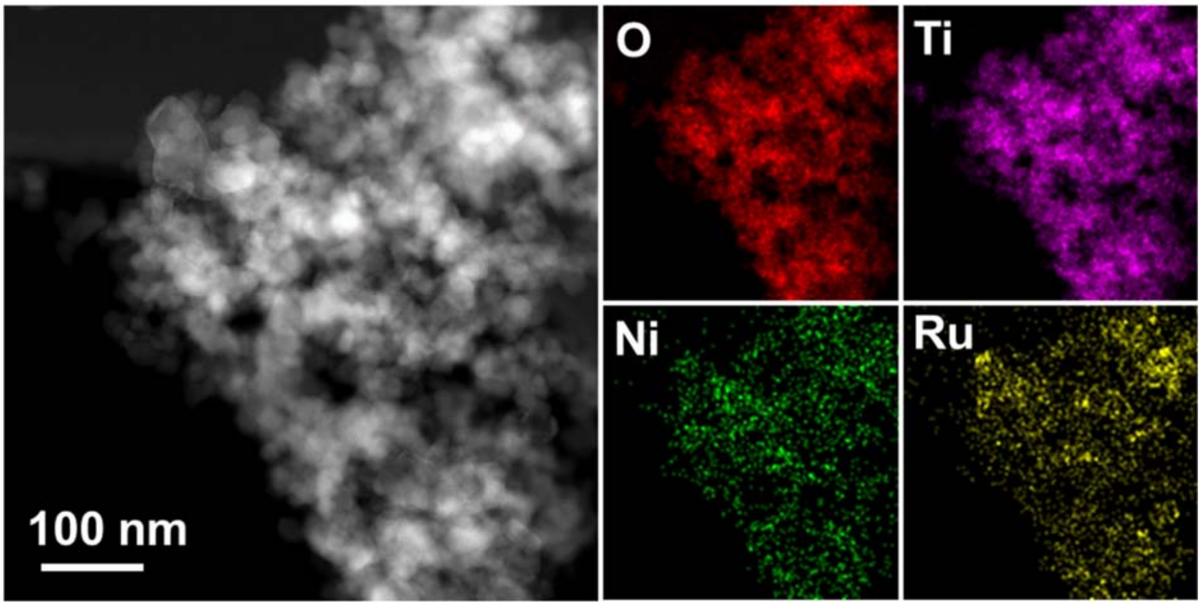

Supplementary Figure 13. Representative low-magnification HAADF-STEM image of calcined 2Ru5Ni/TiO_2 and the corresponding EDS elemental mapping images of O, Ti, Ni, and Ru.

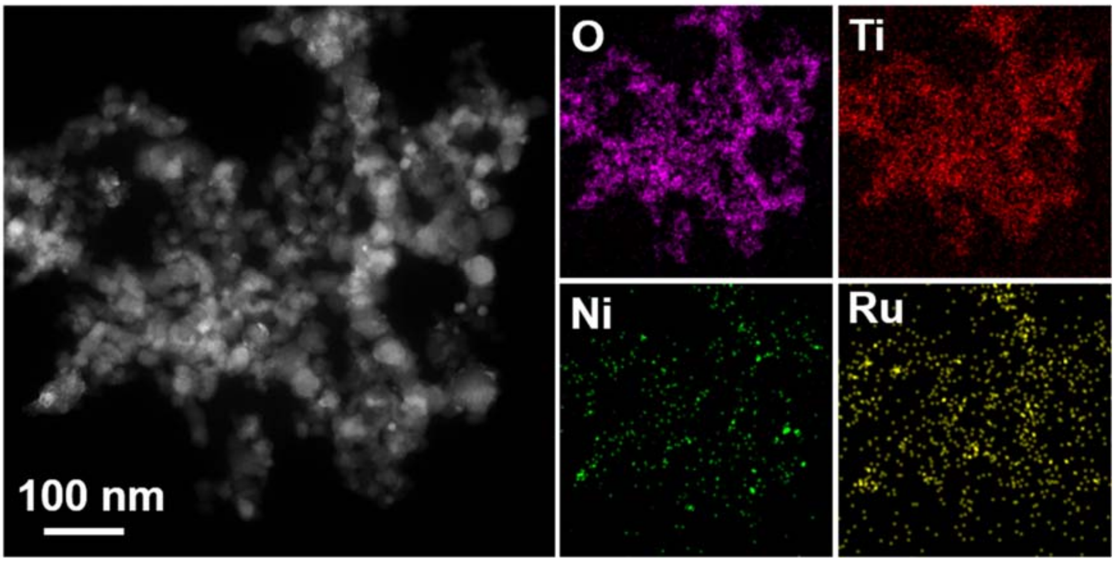

Supplementary Figure 14. Representative low-magnification HAADF-STEM image of reduced 2Ru5Ni/TiO_2 and the corresponding EDS elemental mapping images of O, Ti, Ni, and Ru.

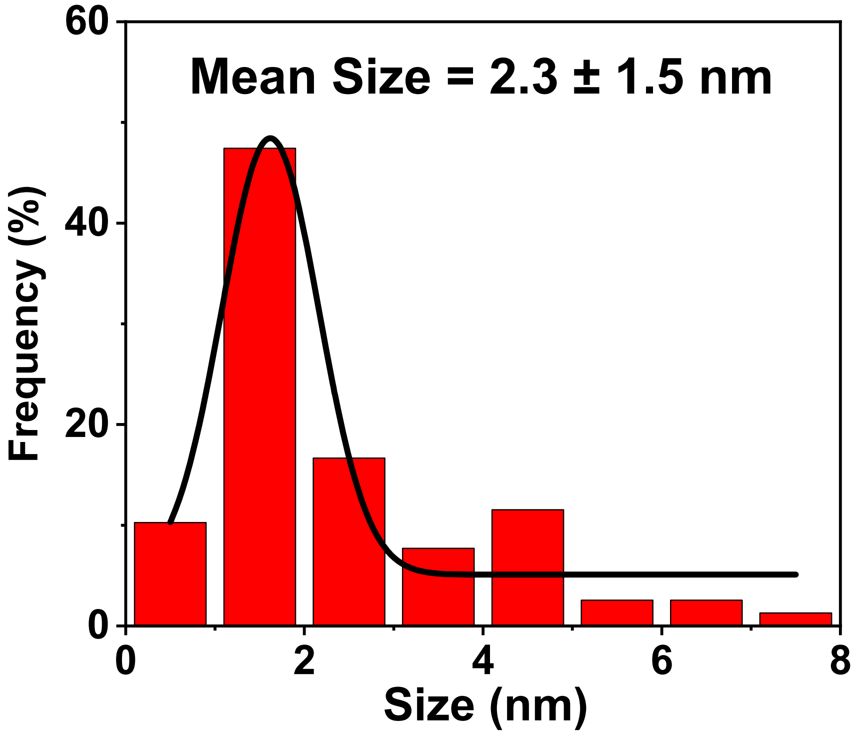

Supplementary Figure 15. Statistics of the Ni species adjacent to Ru particles size distribution of reduced $2Ru5Ni/TiO_2$.

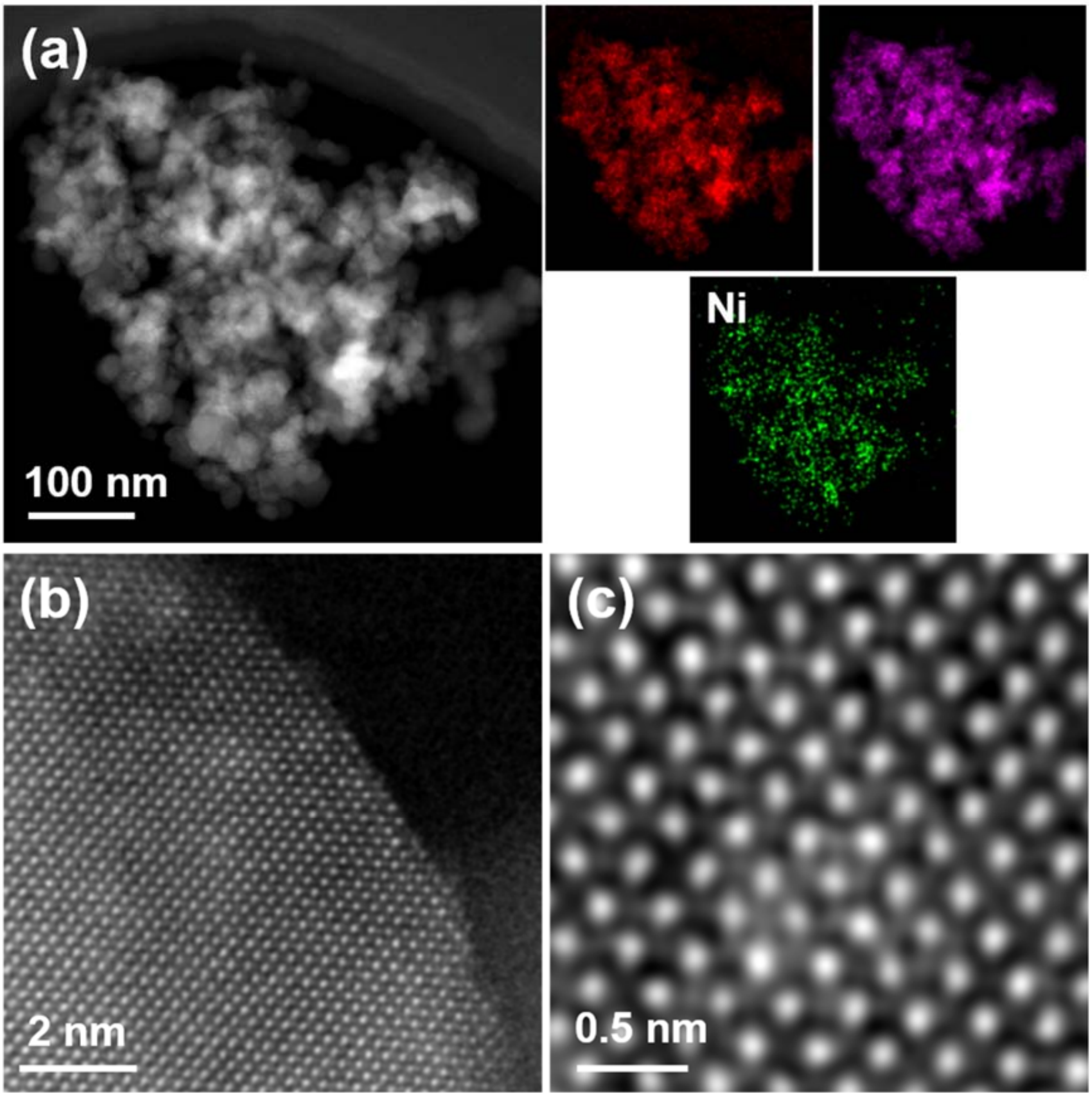

Supplementary Figure 16. Representative HAADF-STEM images of 5Ni/TiO_2 after calcination and the corresponding EDS-mapping images of O, Ti, and Ni. (a) low-magnification (b) and (c) atomic-scale HAADF-STEM images of 5Ni/TiO_2 after calcination.

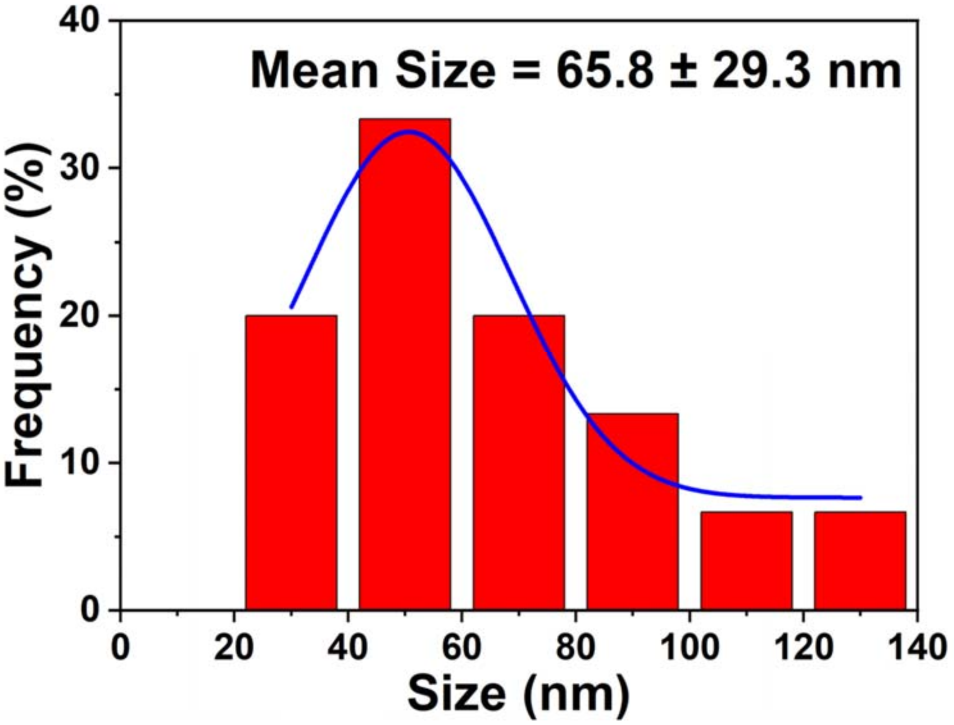

Supplementary Figure 17. Statistics of the Ni particles size distribution of reduced $5Ni/TiO_2$.

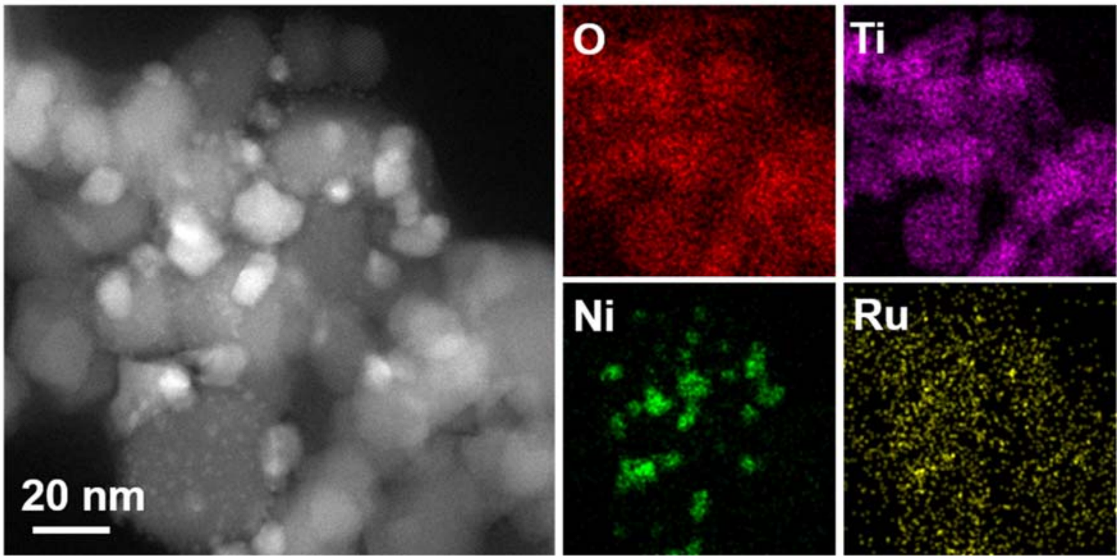

Supplementary Figure 18. Representative HAADF-STEM image of reduced $2Ru5Ni/TiO_2$ and the corresponding EDS spectroscopy elemental mapping images of O, Ti, Ni, Ru.

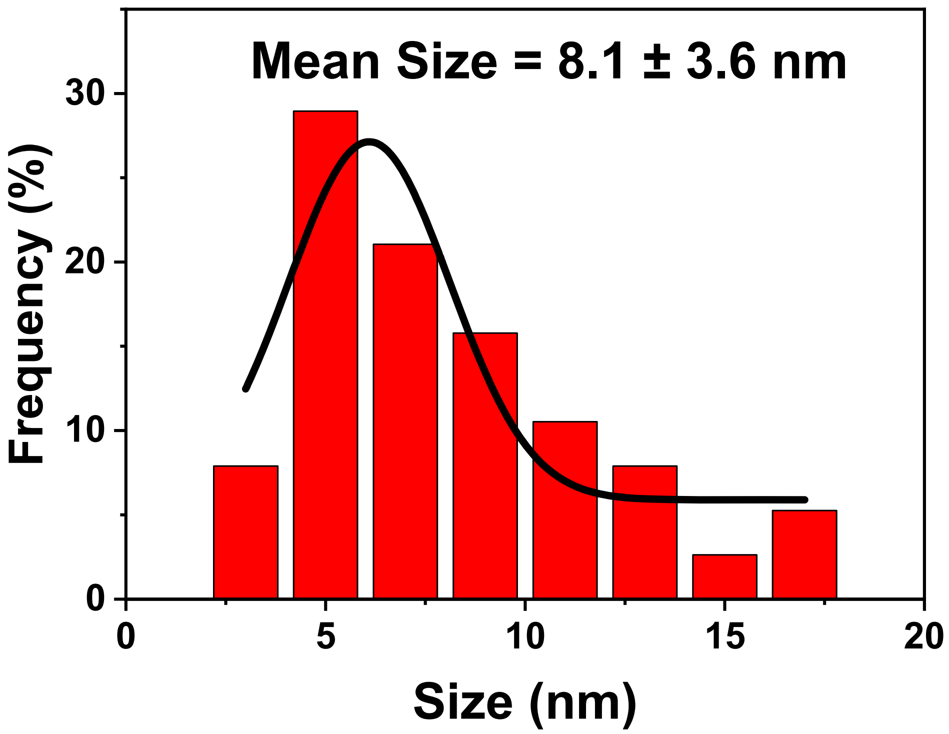

Supplementary Figure 19. Statistics of the Ni particles size distribution of reduced 2Ru5Ni/TiO_2 from Supplementary Figure 18.

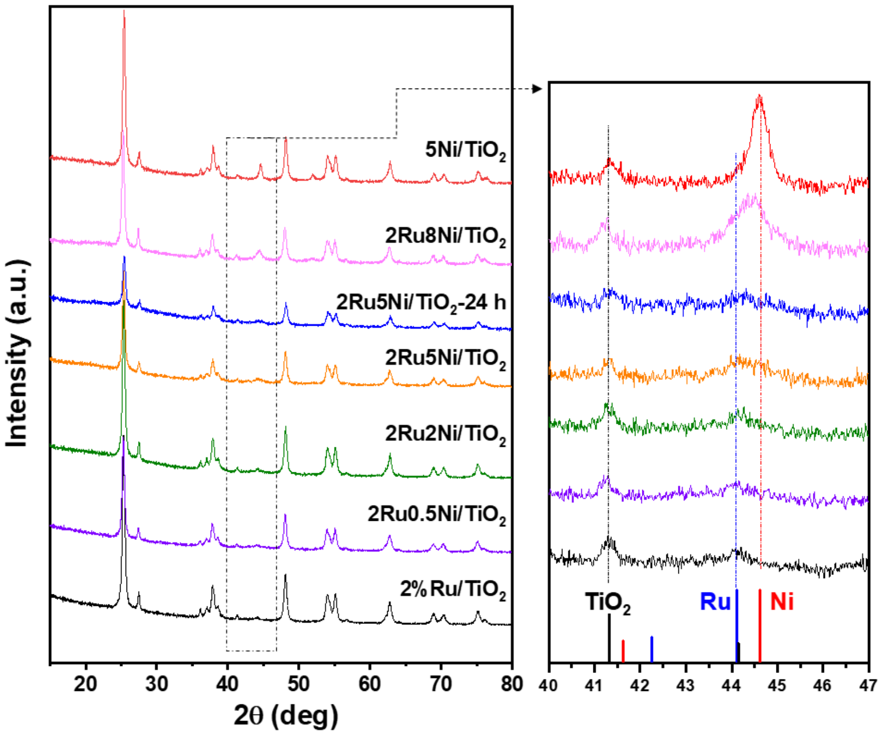

Supplementary Figure 20. XRD patterns of different catalyst in their passivated state. The inset image on the right represents a local view of 2θ = 40 – 47°. The characteristic diffraction signals of TiO_2, metallic Ru and Ni are marked below the inset image.

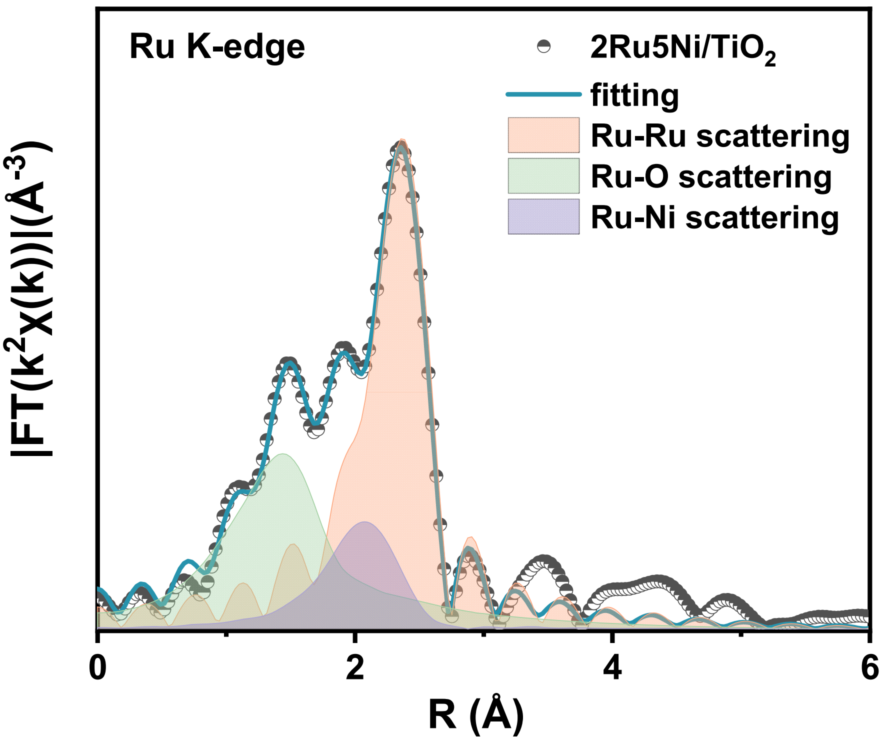

Supplementary Figure 21. Ru K-edge EXAFS fittings in R space of reduced 2Ru5Ni/TiO_2.

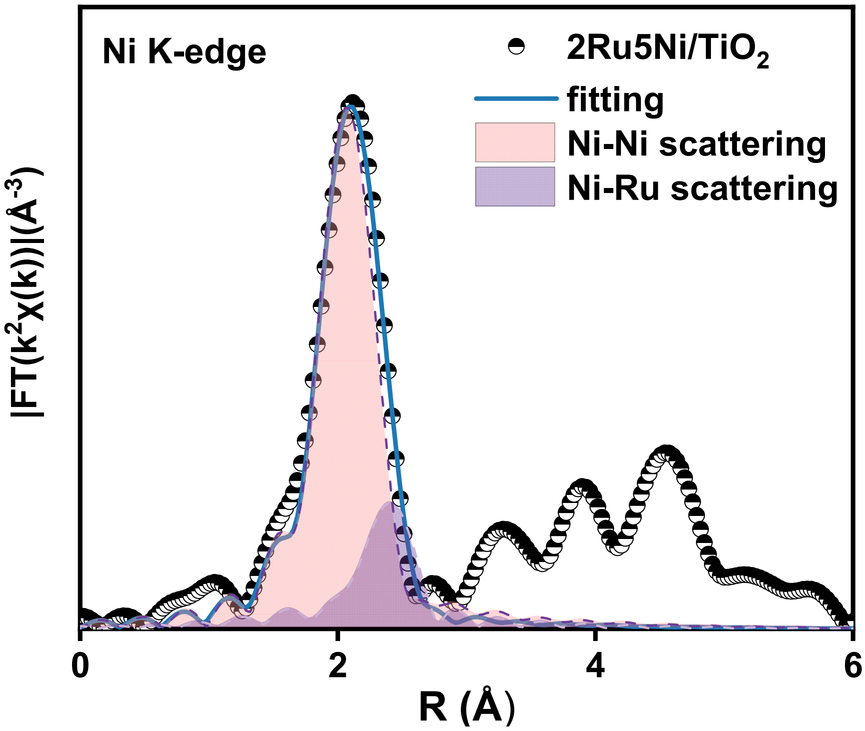

Supplementary Figure 22. Ni K-edge EXAFS fitting curves of reduced 2Ru5Ni/TiO_2.

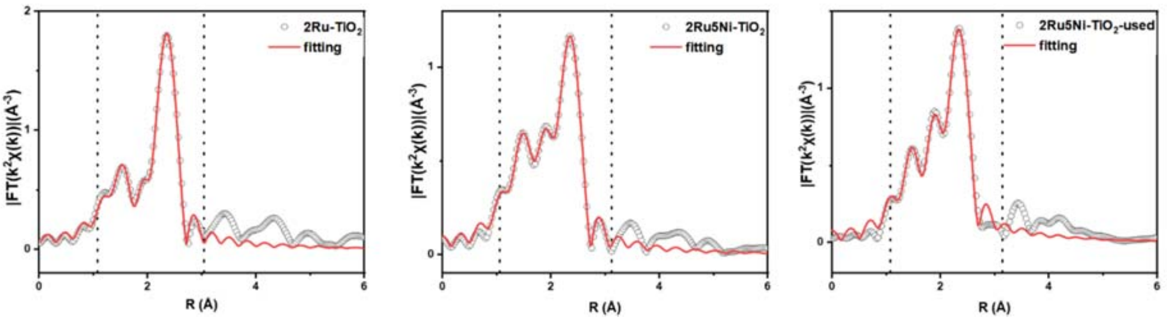

Supplementary Figure 23. Ru K-edge EXAFS fitting curves of 2Ru/TiO_2, 2Ru5Ni/TiO_2 and 2Ru5Ni/TiO_2-used samples.

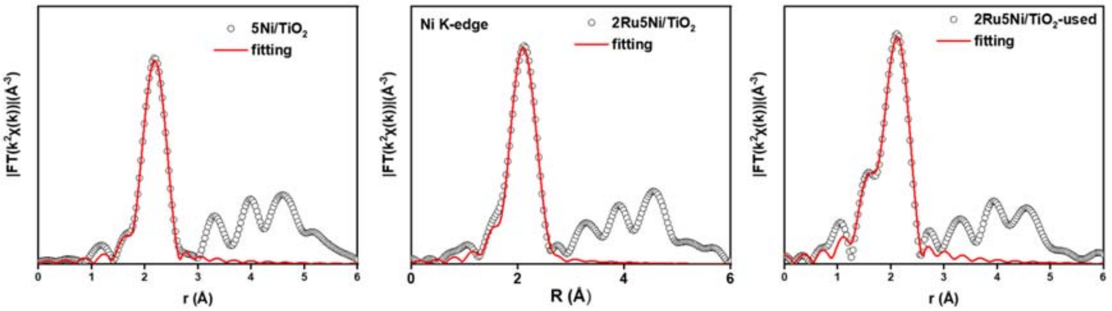

Supplementary Figure 24. Ni K-edge EXAFS fitting curves of 5Ni/TiO_2, 2Ru5Ni/TiO_2 and 2Ru5Ni/TiO_2-used samples.

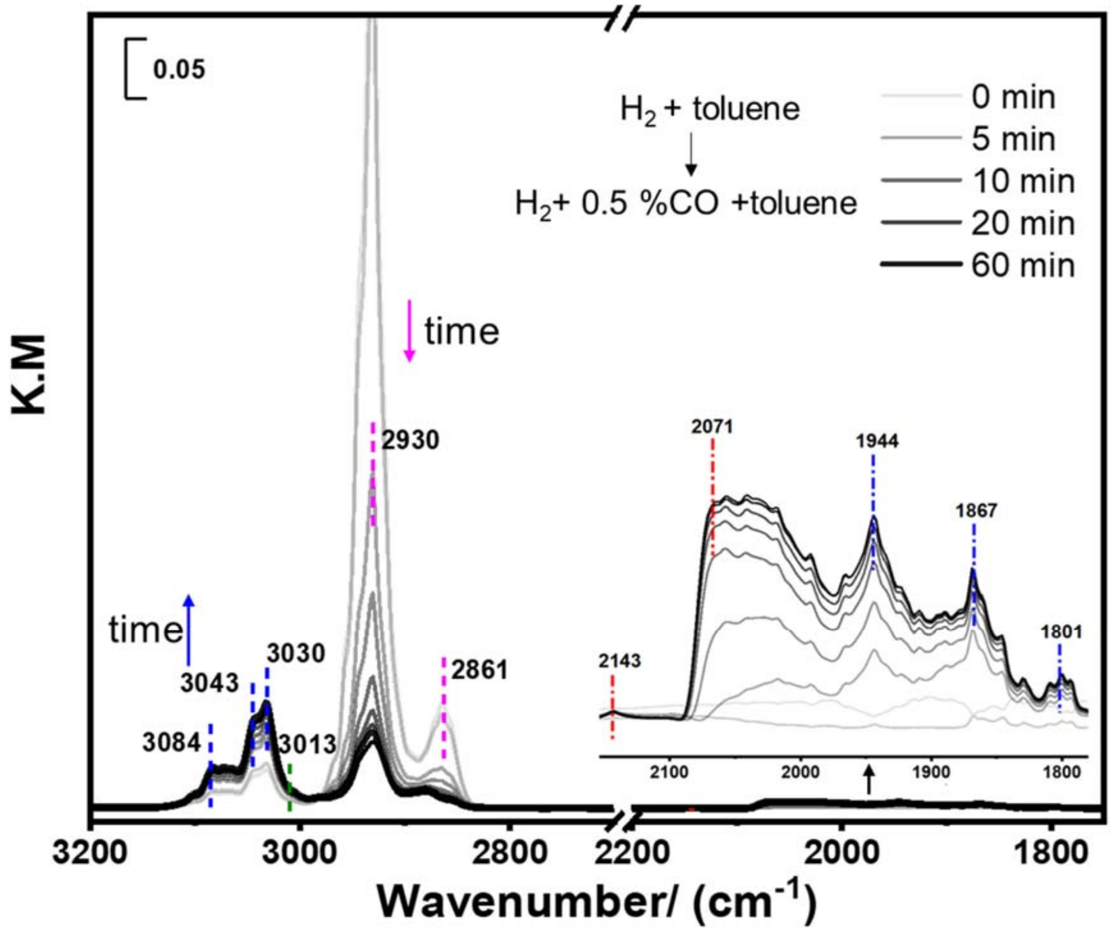

Supplementary Figure 25. In situ DRIFTS of CO-tolerant hydrogenation reaction over 2Ru5Ni/TiO_2 catalyst. The spectrum of 0 min represent the balance state of toluene hydrogenation under pure H_2 in the presence of 0.05 bar toluene. Then the carrier gas was switched to 0.5 %CO/H_2, and the spectra from 0 min to 60 min were recorded. Test conditions: all the velocity of the feed= 20 mL/min, the temperature of the cell was kept at 180 °C.

Supplementary Note 2: It can be seen that under pure hydrogen, most of the toluene was converted to MCH as the intensity of the bands at 2930 cm^{-1} and 2861 cm^{-1} (belong to gas MCH, pink lines marked) were much stronger. However, when 0.5 % CO was added, the bands of MCH dropped gradually but became steady after 20 mins. At the same time, the bands belong to gas toluene (3030 ~ 3084 cm^{-1} and 1944 ~ 1801 cm^{-1}, blue lines marked) increased, while adsorbed CO on Ru (2143 and 2071 cm^{-1}, red lines marked, corresponded to the spectra of **Figure 4**) and gas CH_4 at 3013 cm^{-1} were observed. The spectra indicated on 2Ru5Ni/TiO_2,

CO could weaken the ability of toluene hydrogenation but with CO converted to CH_4, MCH could still be produced, further verifying our CO-tolerant hydrogenation results.

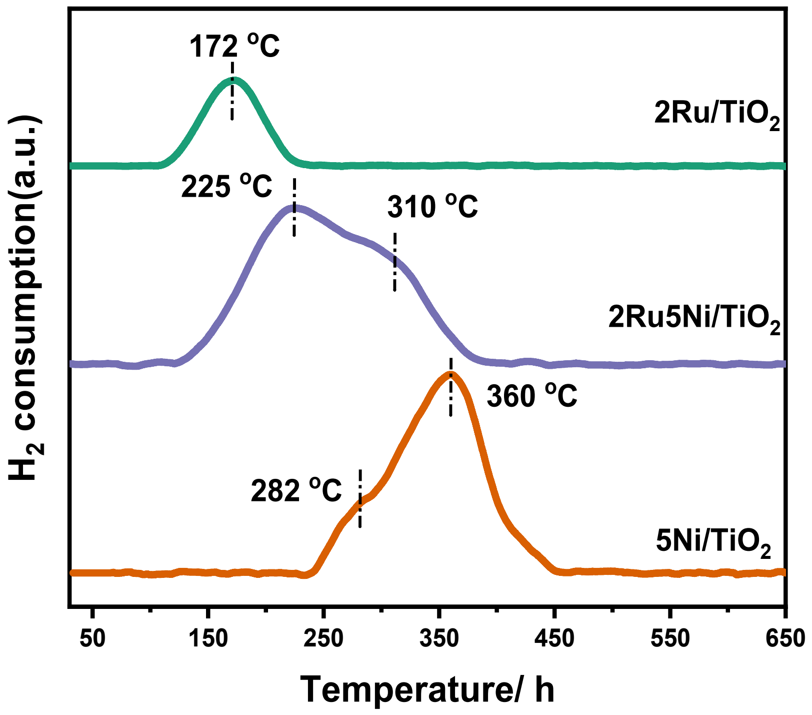

Supplementary Figure 26. H_2-TPR patterns of $2Ru/TiO_2$, $2Ru5Ni/TiO_2$ and $5Ni/TiO_2$.

Supplementary Note 3: For $2Ru/TiO_2$, only one peak attributed to the reduction of RuO_2 centered at 172 °C was observed. For $5Ni/TiO_2$, it shows two hydrogen consumption peaks: the one at ~282 °C is assigned to the reduction of bulk NiO which interacts weakly with TiO_2, and the main peak centered at 360 °C is attributed to the reduction of NiO in strong interaction with TiO_2.[1] Particularly, for $2Ru5Ni/TiO_2$, only two reduction peaks were observed and both shift towards lower temperature at ~225 °C and ~310 °C. The peak started at around 120 °C and centered at 225 °C is dominant, which can be assigned to the overlapped reduction of RuO_2 and bulk NiO, suggesting a strong interaction between RuO_2 and NiO that in agreement with STEM and EXAFS results (**Figure 2** and **Supplementary Figures 21 and 22**).

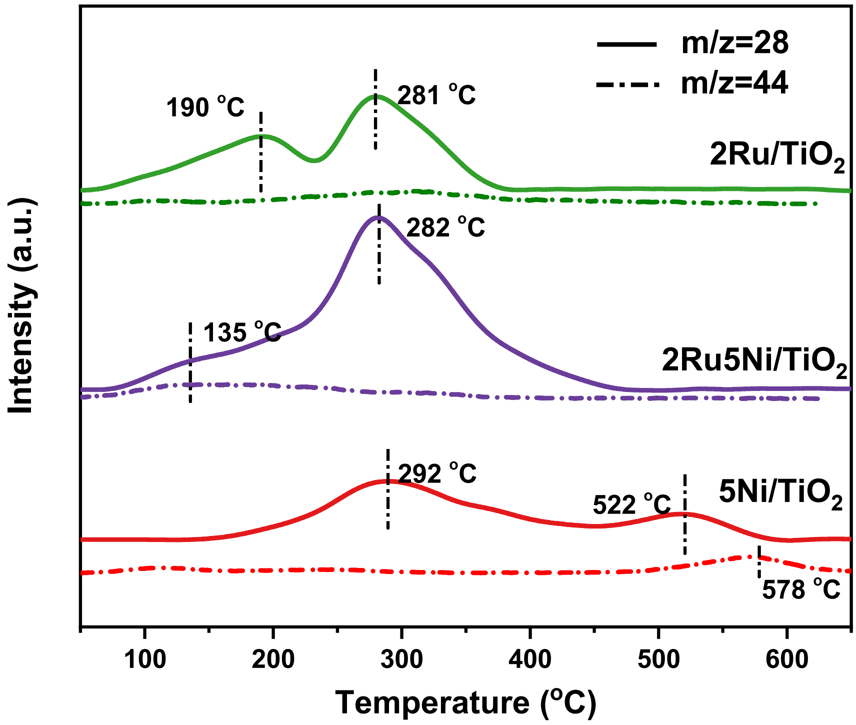

Supplementary Figure 27. CO-TPD patterns of 2Ru/TiO_2, 2Ru5Ni/TiO_2 and 5Ni/TiO_2.

Supplementary Note 4: For 2Ru/TiO_2, two peaks centered at ~190 °C and ~281 °C were observed. The peak at ~190 °C can be ascribed to molecularly CO desorption (m/z=28) on the Ru particles, while the peak at ~281 °C can be assigned to the CO desorption with moderate intensity. Particularly, for 5Ni/TiO_2, besides the peak centered at ~292 °C that can be attributed to the CO adsorption on Ni, the peak at ~522 °C of CO (m/z=28) followed with peak at ~578 °C (m/z=44) appeared. The high temperature of CO desorption on 5Ni/TiO_2 is likely to result from dissociative adsorption of CO, followed by the conversion of CO dissociatively adsorbed to CO_2 via the Boudouard reaction (CO = C+ CO_2) and the other fraction of CO dissociatively adsorbed desorbed as CO.[2-4] However, only two peaks of CO desorption at around 135 °C and 282 °C arise for 2Ru5Ni/TiO_2.The peak at ~522 °C disappeared, revealing the competing CO adsorption for Ru over Ni species on 2Ru5Ni/TiO_2. In addition, the drop of molecularly CO adsorption on Ru from 190 °C to 135 °C indicates the better dispersion of Ru species induced by co-impregnation of Ni, which can be confirmed by EXAFS results (Supplementary Table 3).

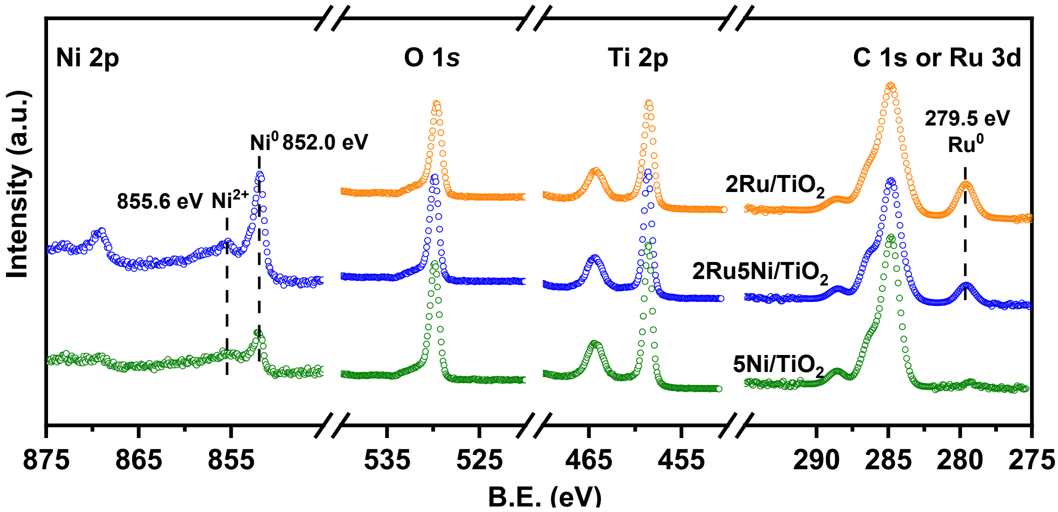

Supplementary Figure 28. XPS spectra of the reduced 2Ru/TiO_2, 2Ru5Ni/TiO_2 and 5Ni/TiO_2.

Supplementary Table 1 Ni contents from ICP-AES results of fresh and used $2Ru5Ni/TiO_2$ catalysts.

Sample	Ni content (wt. %)
$2Ru5Ni/TiO_2$-fresh	4.72
$2Ru5Ni/TiO_2$-spent[a]	4.75

[a] The spent samples were taken after 24 h continuous reaction. Reaction conditions: 180 ^{o}C, 0.5 %CO/H_2, GHSV of the carrier gas = 12,000 mL/g_{cat}/h and WHSV of toluene = 1.4 h^{-1}.

Supplementary Note 5: No nickel loss was found after long-term stability test for $2Ru5Ni/TiO_2$, and the CO adsorption bands observed in DRIFTS results (**Figure 4**) at 2136, 2079 and 2016 cm^{-1} could not match the $Ni(CO)_4$ specific band at 2055 cm^{-1},[5] indicating $Ni(CO)_4$ is not likely to generate during the reaction.

Supplementary Table 2. Catalytic performance of CO-tolerant hydrogenation reaction on different aromatics using a batch reactor [a].

Substrates	Conversion	Products and Selectivity							
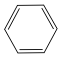 [b]	100 %	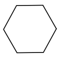	100 %						
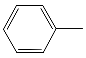	100 %	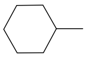	100 %						
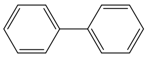	100 %	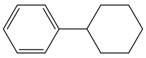	95.8 %		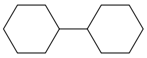	4.2 %			
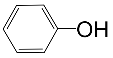 [b]	29.4 %	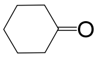	24.6 %		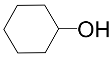	41.0 %		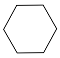	34.4 %
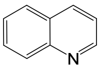	100 %	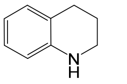	91.6 %		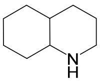	8.4 %			

a. Reaction conditions unless noted otherwise: 30mg 2Ru5Ni/TiO_2, 100 mg substrates, 2 MPa 0.1 %CO/H_2, 3 mL cyclohexane as the solvent, 33 μL n-dodecane as internal standard, 1 h and 180 °C. The total volume of the reactor is 10 mL.

b. 3 mL methyl-cyclohexane as the solvent, instead of cyclohexane.

Supplementary Table 3. The fitting results of the EXAFS spectra of Ni K-edge for $5Ni/TiO_2$ and $2Ru5Ni/TiO_2$ with different operations.

Sample	Shell	C.N. [a]	Bond length (Å)	ΔE_0(eV)	σ^2(Å^2) [b]	R-factor
$5Ni/TiO_2$	Ni-Ni	8.1	2.48	8.8	0.005	0.008
$2Ru5Ni/TiO_2$	Ni-Ni	4.3	2.46	-8.5	0.004	0.014
	Ni-Ru	1.5	2.60		0.004	
$2Ru5Ni/TiO_2$-used	Ni-O	1.0	2.00	-6.2	0.002	0.001
	Ni-Ni	3.8	2.48		0.004	
	Ni-Ru	1.2	2.59		0.007	

[a] Coordination numbers.

[b] Mean squared displacement.

Supplementary Table 4. The fitting results of the EXAFS spectra of Ru K-edge for 2Ru/TiO_2 and 2Ru5Ni/TiO_2 with different operations.

Sample	Shell	C.N. [a]	Bond length(Å)	ΔE_0(eV)	σ^2(Å^2) [b]	R-factor
2Ru/TiO_2	Ru-Ru	5.8	2.67	-4.9	0.004	0.003
	Ru-O	3.1	1.98		0.010	
2Ru5Ni/TiO_2	Ru-Ru	4.4	2.66	-3.2	0.005	0.001
	Ru-O	4.4	2.01		0.015	
	Ru-Ni	1.2	2.55		0.007	
2Ru5Ni/TiO_2-used	Ru-Ru	5.1	2.65	-6.5	0.004	0.01
	Ru-O	2.3	1.96		0.012	
	Ru-Ni	1.4	2.58		0.001	

[a] Coordination number.

[b] Mean squared displacement.

Supplementary References

1. Xu, M., et al. TiO_{2-x}-Modified Ni Nanocatalyst with Tunable Metal-Support Interaction for Water-Gas Shift Reaction. *ACS Catal.* **7**, 7600-7609 (2017).
2. Chen, H., et al. Ru-Cluster-Modified Ni Surface Defects toward Selective Bond Breaking between C–O and C–C. *Chem. Mater.* **28**, 4751-4761 (2016).
3. Zielinski, J. Interaction of carbon monoxide with supported nickel catalysts. *J. Mol. Catal.* **79**, 187-198 (1993).
4. Bengaard, H. S., et al. Steam Reforming and Graphite Formation on Ni Catalysts. *J. Catal.* **209**, 365-384 (2002).
5. Meunier, F. C. On the contamination with nickel and nickel tetracarbonyl during FT-IR investigation of catalysts under CO-containing gases. *J. Catal.* **372**, 388 (2019).
